# Team Role Adoption and Distribution in Engineering Project Meetings

**DOI:** 10.3390/bs10020057

**Published:** 2020-02-08

**Authors:** Kristina Nestsiarovich, Dirk Pons

**Affiliations:** Department of Mechanical Engineering, University of Canterbury, 20 Kirkwood Ave, Christchurch 8041, New Zealand; kristina.nestsiarovich@pg.canterbury.ac.nz

**Keywords:** team communication, engineering project meetings, group interactions, team roles, circumplex

## Abstract

Team communication plays a vital role in engineering management, however, there is a paucity of work that examines how team roles emerge as a response to the communicative processes between participants. This research explored role adoption using qualitative methods comprising observations, questionnaires and semi-structured interviews. Five student teams doing final year projects at a university in New Zealand were observed during the academic year and then interviewed at the final stage of project completion. A number of team roles in the engineering context were identified for students and their supervisors: Explorer; Initiator; Facilitator; Active and Passive Information Provider; Outsider; Active and Passive Connector; Passive Collector; Arbitrator; Gatekeeper and Representative. Personal factors, such as social sensitivity, were correlated with the choice of team behaviour pattern. In addition, the team roles could be arranged in circular order to create a circumplex, the two axes of which were identified as Personal Agency/Communion and Social engagement/Social disengagement.

## 1. Introduction

### 1.1. Context

The behaviour of team members in organisations is crucial to its performance. What does it mean to operate effectively as a team member? It means individuals should do their job and perform team roles in a way that moves the whole group towards the accomplishment of its objectives. However, team communication has the potential to result in misunderstandings, technical disagreements, and conflict. Hence, the roles that members take can affect the team outcomes. The main purpose of communication is to coordinate the collective activities of multiple people.

Inventories exist for team roles, but the question of how and why people adopt one role rather than another is poorly understood. This paper explores the process of team role assignment and distribution among members of engineering project meetings.

### 1.2. Literature Review for Team Role Adoption and Distribution

There are two separate issues. The first is the need to identify the types of roles that exist. This is primarily addressed by various inventories, though, as will be shown, they have different constructs. The second is team role adoption, which relates to the processes whereby individuals intrinsically have preferred roles and adopt those roles rather than others. Related to this is role distribution, whereby role adoption is influenced by the roles taken by others and the needs of the team as a whole. 

#### 1.2.1. Classification Systems for Team Roles (Inventories)

A team role can be defined as the way in which people interact with one another while performing a task in a team [1]. There are two different approaches in the team role literature [2]. The first is anthropological–sociological, with a role as position. From this perspective, a team role is the behaviour that individuals show in relation to their social position and status [3]. The second approach is role as a person, where a role is defined as a combination of the values, attitudes, and behaviour of a person. Roles then emerge from members’ natural preferences [4].

There are many taxonomies of team roles in the literature. The largest number of roles (fifteen) belongs to the Davis taxonomy [5] and the least (four) to Parker [6]. Different sets of roles sometimes overlap. Key differences between the various inventories for roles are the number of roles, focus (on conflict, positive or negative behaviour, formal status), how personality or role preferences are included, the method of data collection of teams’ behaviour (observation, interview), and area of research (business, engineering, etc.). The wider literature is extensive, but the application to engineering is sparse. The main ways of categorising roles are briefly reviewed below. 

Belbin’s team roles

Belbin introduced an eight-role model [7] and defined team role as a pattern of behaviour characteristic of the way in which one team member interacts with another in order to facilitate the progress of the team as a whole. He also later added a ninth role [8] as a result of further research: plant, specialist, monitor evaluator, implementer, shaper, completer finisher, team worker, coordinator, and resource investigator. Each role was proposed to have strengths and weaknesses. For example, plant people were creative, imaginative and could solve different problems, but might be too preoccupied to communicate effectively. Team workers were mild and diplomatic, but sometimes indecisive in critical situations [8]. 

Also, Belbin found that certain combinations of team roles lead to high team performance. This association between team performance and balance of team roles received some support from other researchers [9,10]. A mathematical model was constructed to assign the most suitable roles to team members and, in this way, to create a balanced team with high performance [11]. However, Belbin was also criticized for this team balance idea, as other works found no statistical correlation between team composition and performance [12].

Benne and Sheats’ team roles

According to Benne and Sheats [13], team roles are subdivided into three main categories: task roles, building and maintenance roles, and individual roles. ‘Task roles’ refer to goal achievement, ‘building and maintenance’ were designed to maintain the group performance, and ‘individual roles’ were directed to the satisfaction of participants with their individual needs in the group.

Group task roles [13]: initiator–contributor (proposes new ideas), information seeker (asks for clarification), opinion seeker (asks for clarification of team members’ values), information giver (offers fact or generalisations), opinion giver (suggests, gives opinions), elaborator (offers explanations for already-made suggestions), coordinator (clarifies relationships between ideas), orienter (summarizes the ideas of the team), evaluator–critic (evaluates group tasks), energizer (stimulates team members to act), procedural technician (performs routine tasks), recorder (records group decisions). 

Group building roles [13]: encourager (encourages other members), harmonizer (mediates the differences between members), compromiser (offers compromise in conflict situations), gatekeeper (keeps communication channel open by regulating the participation of team members), standard setter (express group standard), group-observer (record group processes), follower (follows team movement passively). 

Individual roles are typically dysfunctional [13]: aggressor, blocker, recognition-seeker, self-confessor (use group audience to express personal feelings or ideology), playboy (cynicism, bad jokes, lack of involvement), dominator (manipulates people), help-seeker, special interest pleader. 

Comparison between Belbin’s team role inventory and that of Benne and Sheats’

Dulewicz [14] tried to correlate Belbin’s team roles between each other. The results showed a low discrimination between roles, which was attributed to underlying personality factors. In other words, personality traits may be the basic components in a construction of team roles by Belbin [2]. 

Belbin’s inventory assumes that people adopt roles based on their personal preferences, and that in doing so they implicitly consider how their behaviour interacts with that of other team members. In contrast to Belbin, other researchers focused not on a preferred team role but rather on extracted personal styles [13,15]. However, Benne and Sheats’ team role inventory components are based more on the functions of each individual in the team rather than on personality traits that could arise in this situation, as per Belbin. 

Other taxonomies

Another well-known taxonomy of team roles is the team management system (TMS). This was originally developed by Margerison and McCann [16]. Those authors created a model with eight key role functions that were associated with personal characteristics. These roles are similar to Belbin’s as they both made similar assumptions: people choose their team roles according to their personal preferences.

Parker identified four main types of behaviour in teams [15]: contributor, communicator, collaborator and challenger. For example, individuals with a contributor style of behaviour in some situation may adopt a tactical, statistical, specific, measurable, and conservative approach ([15] reviewed by [4]. Parker called his inventory ‘team players style’, and not team roles.

Other researchers created different classifications from existing roles in the literature. This includes the development of a classification of 120 team roles into 10 categories, with each category having a context or ‘essence’ [17]. Then, those authors clustered every team role again into three broader groups: task, social and boundary-spanning roles. Social roles included maintaining the social environment; task roles were about goal achievement and work performance; boundary-spanning roles connect team members with individuals outside of the team [17]. However, there is a problem with this classification system: it is complicated and includes 27 different roles, which are hard to observe and analyze in a real working situation. 

#### 1.2.2. Team Roles in Engineering 

The process of communication in the specific context of engineering teams has been investigated from multiple directions:

- Technical process. Engineering communication has been mostly seen as a technical process that includes software problems [18], communication protocols [19], engineering communication networks [20] and developing electronic communication skills [21];
- Engineering cycle. Information input is defined by the effort or time that engineers need for information assimilation and output is measured by the amount of presentations and the time that engineer needs to prepare for them [22]. The idea here is that that engineers spent more time ‘outputting’ information than ‘inputting’ in different stages of the project [22,23];
- Engineering communication style. There is a difference between engineering communication styles and those from other professions. Engineers tend to use more interpersonal and informal communication channels [22]. The reason for this may be the nature of engineering work, personalities, and different learning styles (listening and discussing rather than observing and reading). Engineers tend to be self-sufficient and use a direct approach in their work [22];
- Communication problems. The main communication problems of engineers have been identified [24], although that work was more of a list than a theory. Communication problems in automotive requirements engineering were identified by [25];
- Engineering communication skills. An emphasis is evident in the literature on the importance of gaining communication skills to work in the engineering industry [26,27];
- Artifacts (boundary objects). Communication in engineering has been studied in many works, such as [28,29,30]. Typical artifacts in this context are white boards, physical models, and computer presentations.

However, for the specific area of types of team roles in engineering, the literature is sparse. The field is dominated by two studies, both from the perspective of business process re-engineering (BPR). This is a type of change-management application, and it is unclear how this applies to other engineering situations. 

Engineering team roles per Platt

In business process re-engineering, it is generally accepted that team members should have various roles for effective task completion. In the research of [31], team roles were identified for the BPR of five manufacturing units (electronic industry) using a case study approach. The data were collected through interviews, questionnaires, group methods and observations. The results showed that the classification of team roles by Platt [32], which is a modification of Belbin’s role taxonomy, was suitable for re-engineering teams. This role set comprises innovators, resource investigators (bring information and ideas from sources outside the group, chairs (team leader), shapers (implement ideas), evaluators, team-workers (promote harmony in the group), organisers and finishers (prevent group from time-wasting). Another finding of this work was the role of leader in re-engineering projects: the leader should provide professional skills and should be able to change their role when moving between different teams if they have missing skills [31]. In summary, those authors showed a method to determine team roles by adapting an existing taxonomy to a specific area under examination.

Engineering Team roles per Nestsiarovich and Pons 

More recently, [33] devised a new categorisation for team roles. The roles were a derivative of those of [13], but further clarified based on an observational study of engineering project interactions. Consequently, several of the roles are identical to those of [13], but others are somewhat different. The roles are shown in Table 1.

That study was based on data from engineering students and industry. The present paper extends this system, based on the observations from a larger dataset.

#### 1.2.3. Existing Theories/Explanations for Team Role Adoption

Ruch [34] proposed a model of team behaviour with several roles: idea creator, information gatherer, decision-maker, implementer, influencer, relationship manager and energizer. Results showed that some roles were positively correlated with job satisfaction and character strengths. They also distinguished current and ideal team roles: current roles depend on the situation, whereas ideal roles were related to personality. There was higher job satisfaction where a current team role corresponded to a person’s ideal one [35]. This is consistent with the theory that people may choose jobs that fit their ideal team roles or craft their jobs towards their ideal role [36]. 

Another group conducted a survey (questionnaire), from which they developed a model of relationship between personality traits, team roles, behaviour and role orientation [37]. It was asserted that different team roles (they used Belbin’s inventory) have different levels of power and influence on society; also, how people see themselves depends on their social positions and on what is expected from them [38]. 

Fujimoto suggested the novel idea to count a ‘role-acquisition frequency’ to study team role adoption processes [39]. In this work, two researchers separately classified the patterns of behaviour of each participant during the meeting time into ten roles. The discussion time was divided into equal 5 min sections, and all behaviour expressions were then identified. The typical length of a discussion was 5–7 sections. A frequency of appearance was calculated for each ten ‘discussant-roles’. They created a three-criterion model based on these parameters and transformed the team roles into a scale system with 11 points [39]. Authors proposed that participants could use this scale to understand which role teams were needed and how they might acquire them to improve group performance. The main limitation of this study is that it was not ‘real-world’ observation but merely a laboratory experiment where people were given tasks to have a 30 min discussion. Main discussant-roles were also received by questionnaire (self-report of people after meeting time) and not supported by observation. In addition, the participants of discussion had equal social status. It could be interesting to see role adoption in teams with different social statuses of members.

#### 1.2.4. Gaps in the Body of Knowledge

The question of how people adopt roles has had limited coverage in the literature. Most of the research literature suggests that team roles arise from individual preferences and the personal characteristics of team members. There is a paucity of work that examines how team roles emerge as a response to the communicative processes between participants. 

Previous authors had a strong focus on formal roles—that is, the way individuals meet job demands [8,40], rather than individual ones. However, the actual behaviour may be a combination of formal and informal roles [41]. Each individual can be identified with at least three types of roles: formal (job task), informal (personal content), and dramaturgical (created in interaction between members–actors) [41]. The last type of team role—dramaturgical or role as communication interaction (protagonist, antagonist, team member, auxiliary and audience) [42]—was not widely studied or adopted by others. 

Another problem is that most research about team roles relied on self-assessment, participants’ assessment, and less often on peer rating or personality tests, whereas only a few studies used observation as the main method of data collection [43]. 

There are a number of gaps in the literature about engineering team behaviour, and these are potential areas for further research:

- Effect of status. It could be interesting to see how the presence of formal leaders influences the participants’ behaviour. What is the difference between communication with a manager/ supervisor and without?
- Multidisciplinary team composition. Engineering problems often involve inter-disciplinary teams, hence a non-homogeneity of professional background. There appears to be little or no research into how different disciplines influence the participants’ behaviour, or what factors contribute positively or negatively to team performance in these situations;
- Role assignment. There is a shortage of studies about team role assignment that are based on observations in engineering teams. It is the observation of the present authors that engineering communications tend to be characterised by the high importance of visual artifacts in communication, strict time frames and regularity of project meetings. There may also be large differences in professional expertise, resulting in differences in communication style. Use of specific technical terminology may contribute to misunderstandings. However, the effects of these variables have not been formally reported in the literature. Most studies in the area of engineering communication have a strong focus on performance, boundary objects (artifacts) and personality rather than team roles. Finally, there are several areas where the literature is sparse, and which could benefit from further research. One of these is to develop a better understanding of the process of team role adoption in engineering teams. It would also be useful to better understand the reasons behind role adjustment;
- Stability of team roles. There are unexplored questions about the stability of team roles and how team roles change over time.

The current paper focusses on the question of role assignment. 

## 2. Materials and Methods

### 2.1. Objective

The aim of this work was to develop a conceptual model of casual role assignment (adoption and distribution) in the engineering context. Specifically, the aim was to identify how participants of engineering project meetings choose and acquire communication behavioural patterns.

### 2.2. Approach

The current work continued with the framework of Nestsiarovich and Pons, which is based on the Benne and Sheats’ inventory [13]. We selected that inventory instead of Belbin’s, because we had observed that team role assignment was, in practice, primarily based on functional rather personal preference, at least in engineering teams. 

Qualitative research (QR) methods were used in this study, because the data were mostly of a qualitative nature (behaviour of people at the meetings and their team role). We combined multiple data collection methods (observation, interview and questionnaires) that allowed us to evaluate engineering communication from different perspectives. This was similar to the approach taken by [31].

In the first phase, an exploratory study was conducted using only the observational study method. This was used to refine the observational method and to identify a preliminary set of team roles. In the second phase of the study, we added a semi-structured interview, a questionnaire and a Big Five personality test to the regular observations to collect more data from participants. The current paper primarily reports on findings from the data collected from the second phase.

We investigated communication in teams of engineering students who were doing a final year project. A total of five teams participated, each comprising, nominally, four students and one academic supervisor. The number of teams was determined by what was feasible for the researcher to follow since the teams tended to all meet on the same day. Students were from the University of Canterbury, New Zealand. All students were in the final year of a four-year Washington Accord [44] engineering degree. 

Selection criteria for inclusion of project teams in the study were:

- The group consists of 3–8 members;
- The group meets on a regular basis;
- The group includes at least one participant from a different engineering discipline (official position or education) than other team members, or;
- The group includes at least one person with higher official position than other members, which was usually the supervisor or a postgraduate student;
- Project discussion is in the initial stage of development (first five meetings);
- All members agreed to participate.

Data were collected via initial questionnaire, observational study, and semi-structured interview, as follows:

Initial questionnaire. We gave a small questionnaire to participants (four questions) to identify their education background, gender, and social links between members of the group (see Appendix A). Also, we used the ‘50-item IPIP version of the Big Five Markers’ test created by [45] and taken from [46]. We selected this test and online resource because of the limited numbers of questions (participants needed only 10–15 min to answer).

Observational study. Students had regular meetings with their supervisors, typically once per week at university and occasionally at external organisations (with clients). During the meeting time, the team discussed project progress and problems that arose, reported the results to their supervisor or client, and asked questions. The researcher observed team meetings on a regular basis, typically weekly, during the whole academic year. The researcher did not participate in the discussion but sat aside taking notes. There was no audio or video recording, only written notes using the previously developed interaction diagram (ID) method [33]. Participant identity was recorded using a code. This helped to track the longitudinal observation across multiple team meetings. 

Semi-structured interview. At the end of the observation period, a semi-structured interview took place with each participant to clarify communication situations and team behaviour. Questions are shown in Appendix B. 

Ethics approval was obtained from the University of Canterbury Human Ethics Committee (HEC 2017/70/LR-PS), and consent was obtained from all participants before commencing.

### 2.3. Data Processing Methods and Sequence of Data Extraction

#### 2.3.1. Communicative Approach

We followed the communicative approach developed by [43]. This suggests that team roles are produced and shaped in communicative behaviour, through interaction patterns. These casual behaviour patterns (spontaneous reaction to communication situation and personal attitude to other team members) are opposed to the expected team roles that arise from job duties (official status).

#### 2.3.2. ID Methodology 

We previously developed a method to record team interactions using a paper notebook, hence avoiding the intrusiveness of audio and video recording [33]. This was done by preliminary observation of students’ interactions at several engineering project meetings. This is called the interaction diagram (ID) method.

The ID method is less invasive for participants than an audio or video-recording and can be used in a time-pressured situation. It also makes the process of receiving ethics approval easier. By using the ID method, the researcher does not need post-event data transcribing, which can be time-consuming. 

**Figure 1:**
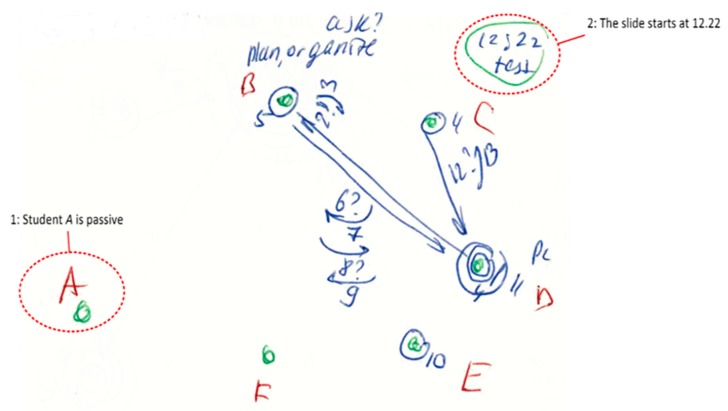
Interaction Diagram: communication situation 1 [reproduced from [33] by permission].

In the current study, communication patterns were recorded by the ID method as a set of notes or phrases. Most of the team roles were identified from this qualitative data. The combination was subsequently post-processed using QR methods. Below (Figure 1) is an example of notetaking. 

#### 2.3.3. Sequence of Data Extraction 

Notes and symbols on interactions diagrams were used to extract data about participants’ behaviour to the research journal (after every meeting). Typical behaviour patterns (keywords) were underlined and grouped in the research journal using NVIVO software (version 12 Pro, QSR International, Melbourne, Australia) for qualitative analysis. 

A number of team roles was assigned to each participant of the project meeting. Our approach used the identified roles as defined by Nestsiarovich and Pons [33]. We adopted the general approach of [31] for the way that team roles were inferred. 

Data about team roles for the whole period of observation were summarized and compared with interview answers (self-reports). In the case of inconsistent results, we trusted our observations. The final data were then analysed for communication progress and problems in the teams. 

**Figure 2:**
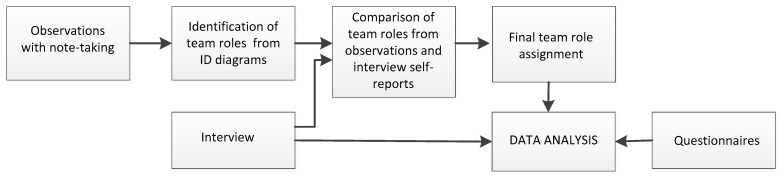
Sequence of research methodology.

The flowchart below depicts the sequence of the research methodology (Figure 2).

## 3. Results

### 3.1. Summary of Participant Demographics and Nature of Projects

Twenty-five participants in total took part in the study (five teams with five participants in each). Every team consisted of one supervisor and four students. Among the participants, 21 were males and four females (one supervisor and three students). 

Students were of the same age group—20–25 years old—and from mechanical or mechatronic areas of engineering. Most of them (all groups except team 4) did not know each other before the final year project started.

Students conducted their project as a final year work. These were large projects that needed the whole academic year for completion. Each team had some engineering problem to solve according to the brief providing by an external client. Participants generally had one or two official meetings per week and there could be also other kinds of communication between them. However, the focus of our research was on the official project meetings of students and their supervisor/client that took place at the university or in the external organisation.

### 3.2. Observational Data: Refinement to Previous Team Role Classification

The second stage of observations of student teams gave more information about participants’ behaviour. We found that the previously identified list of team roles was not complete and needed some adjustments. Thus, we found that the *Information Provider* can be active or passive, and even neutral. By active *Information Provider* (we consider this as a subrole), we mean a behaviour when participants collect all information actively, e.g., give report or instructions—a typical behaviour of a student team leader or supervisor. Passive *Information Provider (Passive IP)* is a type of behaviour when people provide information in response to some external communication impact (give an answer). 

The same was found with the role of *Connector*. We originally supposed that this role was a passive one, because people working with emails cannot actively participate in the project meeting at the same time. However, several observations showed that such connections with outside-of-team people through video or audio conference (by telephone) requires active participation and involves communication processes for connectors. Therefore, *Connector* was also divided into two subroles: active and passive.

### 3.3. Interview Data

All participants were asked 14 questions about their team communication in a written form (see Appendix B). Five questions were about team roles. 

#### 3.3.1. Self-report of Team Role Adoption

Two questions specifically addressed participants’ own assessment of their team roles. Participants first described their own intuitive perception of their communication style (using free text) and then chose team roles from a list. The list of suggested roles contained a description of typical behaviour patterns (see Table 1). 

[Question 5] What is your intuitive perception of your own communication style in this team?

[Question 6] Please tick all team roles (communication patterns) that you think describe your typical communication behaviour. 

Quantitative data from Question 6 are presented in Table 2.

Table 2 shows that the most popular chosen team roles among observed participants were *Initiator*, *Explorer*, *Facilitator* and *Gatekeeper*. The least popular were *Outsider* and *Passive Collector*.

A possible explanation for this is as follows: even if this study considered only casual team roles, *Facilitators* among students were sometimes elected by other group members. *Initiator* (provides new suggestions) and *Explorer* (asks questions) were popular among active students and supervisors, which comprised the majority of participants. This is possibly because completion expectations elicit active communication behaviours from participants. It was observed that *Passive Collector* and *Outsider* were chosen by the fewest participants. 

Each answer from the interview was compared with observational notes. Interestingly, team roles chosen by participants were not always the same as, and even sometimes opposite to, what was observed by the researcher (e.g., a very passive participant considered themselves being quite active and asking many questions, whereas in fact they asked only 3–4 questions for the whole academic year). This can perhaps be attributed to egocentric attitudes. Social comparison theory [47] suggests that we evaluate ourselves by comparing ourselves with other people that we believe are similar to us. When we assess our behaviour by others’ reaction and estimation, there is the potential to be biased towards a favourable assessment [48]. 

The *Gatekeeper* role is worth commenting on further. A *Gatekeeper* regulates communication flows, addresses questions or comments to participants that are inclined to be passive and tries to keep members from dominating the communication.) The first comment is that the popularity of the *Gatekeeper* role was somewhat unexpected. Perhaps participants may have anticipated that regulation of communication flow was important for the good organisation of discussions, and hence participants may have focussed their agency towards this. However, the second comment is that observation data often contradicted the interview results. Participants whose behaviour was similar to gatekeeping sometimes failed to recognize they had adopted this role. On the contrary—sometimes participants who thought of themselves as acting as communication regulators did not actually behave in this way. Possible explanations for this might be a wrong understanding of the role, biased self-perception [48], or faking good.

The results of the interview showed that sometimes participants would be happy to adopt different team roles but could not do it for some reason. A typical reason was these roles being occupied by other group members: *‘If other people covered information there may be no reason for me to contribute’*. 

#### 3.3.2. Adjustments to Communication Behaviour

Whether communication behaviours change, and why, was the subject of Question 7: *‘Do you feel that you changed your communication behaviour at different meetings? Which communication situations caused that?’*

The majority of students (17 out of 20) reported changes in their communication behaviour in different circumstances. The causes may be summarised as follows:

- Presence of supervisor or client/boss (two students);
- Chairing a meeting or not (two students);
- Less personal progress in project tasks, unprepared meetings or relatively unknown topic lead to low desire to contribute in discussion (three students);
- Some students were sensitive to negative critique, and hence intended to be passive (two students);
- Other students became active when they felt that a team or person needed their active contribution (‘*At some meetings where there was a talking point that was getting stuck I tried to shift the conversation’, ‘When one of our team members was away, I filled the role of Information Provider*’) (four students);
- Confidence: ‘*throughout the year I gained more confidence in the work I had completed*’ (two students);
- When participants felt tired, unwell, or were just in a bad mood, they were less likely to be active (two students).

As for supervisors, changes in their communication behaviour at meetings apparently depend on students’ project progress or client needs: *‘I became more assertive half way through when the client had expressed a concern regarding team achieving goals’, ‘I changed communication style when there were unsolved problems or slow progress in the team’*. 

#### 3.3.3. Suppression of Communication by Activity of Others

Question 8 addressed the extent to which contribution was inhibited by others: *‘To what extent do you feel that other people’s discussions prevented you from making a contribution at meetings?’* Most students (16 out of 25) mentioned little or no prevention from making a contribution at meeting. However, several participants complained they were unable to express their ideas because of the excess activity of others: *‘Some members would talk for the duration of the meeting, and as a result I was unable to express ideas‘*. This is consistent with the concept of production blocking. Generally, it was other students who were dominating the conversation, and some students themselves admitted to being too active. One respondent mentioned the style of a supervisor that ‘*did not allow me to express ideas’.*

According to the observational data and data from other interview questions, there were no *Gatekeepers* in those teams. A *Gatekeeper* helps to balance the communication flow and to distribute communication turn-takings between team members. If this role (casual or official) is absent, then some participants may be too active or too passive.

#### 3.3.4. Physical Location 

For each team, the meeting location tended to be the same throughout the year. Question 13 explored the extent to which location affected communication: *‘Did you feel that location of the meeting and your position inside the room predefines your communication style?’*

Half the participants mentioned that location in the room was not important for them at all. The other half had something to say about position. Most participants preferred to sit in front of the person they were talking with. Some students mentioned that a round table created a sense of having an equal opportunity to talk. One student complained that meeting in the supervisor’s office did not make them feel comfortable: *‘I associate the office negatively and did not enjoy being there’*. To summarise, physical location in the room may be also considered as one of the factors that determines communication style and perhaps even team role adoption.

### 3.4. Table of Team Roles. Main and Secondary Roles

During the academic year, we observed multiple types of behaviour for each participant. If some behaviour patterns were repeated several times during the meeting, we assumed that this person adopted the relevant team role. At every meeting, the team roles of participants could be the same or vary significantly. Apparently, this depended on the communication environment of the meeting: quantity of people, level of discussion, physical location, and other factors. Some of these factors were quite obvious and it was easy to recognize them (e.g., the presence or absence of supervisor), others were difficult to know, such as personal circumstances, mood and health.

Combining information from interviews (Questions 5 and 6) with observational data, we chose, for each participant, the most frequent four roles: two main roles that were the most obvious in their repetitive use (or just one, mentioned twice), and two secondary roles that appeared less often or in special circumstances. 

The resulting team roles assignment are in Table 3. Bold type denotes supervisor. According to our observations, all supervisors had *Facilitator* as the first main role, perhaps because of job duties. Letters from A to E represent coded names of the participants and number represents team, for example, 1B—participant B from team 1.

As mentioned earlier, the team roles chosen by participants themselves were not always the same as assigned to participants by researcher. In the case of controversial results, we trusted our observations. 

### 3.5. Role Assignment and Team Needs

An aggregation was made of the various data:

- Team needs were inferred by observation;
- The personality of each team member from the Big Five personality test. Here, we only report on the Agreeableness variable;
- Personal attitudes were determined from the question ‘How comfortable did you feel in this team communication?’: 10—very happy, 9—happy, 8—good, 7—satisfied, 6—not satisfied, 5—unhappy, 4 and below—very unhappy. This primarily addresses aspects of feeling of participants.;
- Main and Secondary team roles were as identified by participants, informed by observation (see above).

The results are shown in Table 4. 

Bold type represents supervisors of the team, whose main team role—*Facilitator*—is fixed by their official position. Green colour highlights a team role that corresponds to team needs, as seen from interview and observation data (column ‘Team needs’ in Table 4). For example, according to the observations and interview data *‘I felt disengagement of some team members’*, Team 1 needed *Gatekeepers* and *Connectors*, and participant 1D took the role of *Gatekeeper* from time to time, that is, they responded to team needs. Apparently, this was not enough, and the team needed more *Gatekeepers*.

Summary of the team role assignment:

Team 1. As seen in the observational data and interview, team 1 had some communication problems with their client: ‘What I did not like is our communication with clients. They always change their mind and students were frustrated. That was not the students’ fault’. Hence, the team needed a role of Connector.

Also, according to the interview answers (*‘I felt disengagement of some team members’)*, the team needed someone who could regulate the communication flow, involving passive students in the communication—i.e., a *Gatekeeper*. Table 4 shows that these roles were fulfilled only partially, not on a regular basis (secondary team roles). Participants 1A and 1D, with relatively high agreeableness (40 and 45), sometimes became a *Connector* and *Gatekeeper*, whereas 1B, with low agreeableness, remained passive all the way through the project and did not respond to the team’s needs. 

Student 1C was unhappy with the team communication because *‘People not listening well, poor recollection of previous discussion; laziness’*. They felt themselves to be a ‘*driver who pushed others’*. According to the observations, they took roles of *Facilitator* and *Representator*, however, they apparently would prefer other people being more active. As evident from Table 4, this student had quite high level of agreeableness (social sensitivity), which is possibly why they followed the team’s needs for organising and pushing, rather than their own preferences.

Team 2. From observations, only one student (2A) in team 2 was very active. This student was not happy with the team communication and thought that other team members had a *‘Lack of regular engagement and involvement in all workstreams’*. However, according to the other participants in this team, their passiveness might have arisen from the over-activity of 2A: *‘I felt that other people’s discussions prevented me from making a contribution at meetings due to conveying of their own ideas’*. 

Team 3 apparently had some problems with communication. According to the observations, there was much misunderstanding between team members. Participants, both supervisor and students, were not happy with the project meetings. The supervisor complained that the team was not good at planning and self-organising: *‘Sometimes I really had to ask what the individual meant when they make a statement (vague). I often had a feeling that the team was unclear of their actions. They seem to be reactive in communication’*, whereas students were convinced that the supervisor ‘*did not understand the project’* and the style of supervision prevented them from active participation: *‘It seemed like I would get attacked at meetings, so I stopped speaking unless I had to’, ‘He seemed like he had no interest in the project from the get-go’, ‘A lot of time was wasted on things we did not think was necessary but supervisor did’.*

From the perspectives of team roles, this team lacked a person among the students who could facilitate (*Facilitator*) the project alongside with the supervisor—organising and distributing duties between students. The role of *Arbitrator* might also be beneficial in such situations.

We think these communication problems cannot only be explained by chosen role. There may be other possible reasons. For example, as the Big Five test showed, both students of this group and the supervisor had low levels of emotional stability and social sensitivity (except 3C), that might predispose them to conflict situations from the beginning. That is consistent with the literature that shows that low emotional stability and agreeableness leads to low satisfaction with team communication [49]. Another possible explanation is that the nature of the project did not provide sufficient role opportunities: *‘The testing part of the project could have simply been done by one person which made it harder for us four to split the work evenly’.*

Team 4 seemed to have good communication and participants were happy with it. The social sensitivity of team members was high as well. The only problem was that one or two participants tried to monopolize the discussion. In other words, there were too many active roles in the group (*Initiators*, *Active IP*s, *Explorers* and *Active Connectors*), and therefore more roles of *Passive Collector* (only the supervisor took it regularly) and *Gatekeeper* might be beneficial here.

Team 5. The main problem of team 5 was that it consisted of people with different levels of technical knowledge and different understandings of the project. Thus, according to interview data, student 5B felt a lack of confidence due to limited experience in the technical side of the project, and also comprehension difficulties. According to the observational data, most participants (4 of 5 including supervisor) were very passive and did not interact much during the project meeting time (almost no discussion, no questions). Only one student, 5E, regularly took the active role of *Initiator*. 

## 4. Discussion

### 4.1. Dependence of Satisfaction on Social Sensitivity

Social sensitivity is an aspect of empathy (personal ability) that helps a person to understand the feelings of others in a group [50]. A previous study on students performing long-term research showed that team sensitivity was highly correlated with project team performance [51]. It is one of the most important factors that helps a team to successfully perform a variety of tasks [50]. Team members with high levels of sensitivity to team needs find it easier to trust each other, and they are not afraid to share their opinion. In situations with miscommunication, such people keep their focus on problem-solving, rather than starting personal conflicts. Social sensitivity has been found to be even more important than general intelligence [52]. The *Agreeableness* attribute of the Big Five personality traits can be taken as an approximate measure of social sensitivity. It captures some aspects of how well a person interacts with other team members and responds to team needs. The maximum score for *Agreeableness* was 100 points, minimum was 0. We selected 50 and 35 as the cut-off points for a descriptive scale of high, middle, and low agreeableness. 

Based on our findings, we suggest that social sensitivity is a key variable for team members to feel satisfied in the communication experience. We simplify this to four combinations of social sensitivity and communication satisfaction:

1. High social sensitivity (*Agreeableness* over 50) and low satisfaction from team communication (unhappy, satisfied). Participants may accept a role that is needed in this team; however, they are not happy with the communication processes because their roles are not consistent with their individual objectives and expectations;
2. Low social sensitivity (*Agreeableness* below 35) and low satisfaction from team communication (unhappy, satisfied). Participants follow their own ideas and preferences in communication, however, there are some problems in the team (or personal problems) that cannot be solved by this;
3. High social sensitivity (*Agreeableness* over 50) and high satisfaction from team communication (happy, very happy). Participants may accept the role that the team needs and this is consistent with their individual objectives and expectations, so they feel happy. However, the operational needs of the team may not be met;
4. Low social sensitivity (*Agreeableness* below 35) and high satisfaction from team communication (happy, very happy). Participants follow their own ideas and preferences in communication, and this apparently makes them feel satisfied with the team communication. However, the operational needs of the team may not be met.

The combinations of team satisfaction and the social sensitivity of the team are in Table 5 (the green colour represents the best combination) along with our proposed descriptive summaries. 

On its own, this model presents difficulties. The first is the obvious problem that, if satisfaction depends on social sensitivity, which in turn is an agreeableness character trait, then teams that lack members with the necessary trait are going to be in difficulty. The trait models of personality suggest that such attributes are relatively fixed, and, while not immune to change, will only change slowly. The model suggests that such teams will tend to either not get the work done to the same standard (which has adverse implications for the client of the engineering project) or will have low satisfaction (which has adverse implications for the manager of the project). We tentatively suggest a solution, which is for project managers to encourage social sensitivity in meetings, perhaps via the setting of behavioural expectations and an organizational culture. This is a call for engineering managers to exert leadership in the way they shape the organizational culture of their subordinates. 

The second difficulty with the above model is that satisfaction relates to what participants feel, and this is not the same as project performance. People can feel personally satisfied, even while a project fails. To address this, we needed to introduce an attribute of quality of outcomes. This in turn resulted in a different conceptualisation of how team roles are adopted, as shown below. 

### 4.2. Model: Circumplex of Team Roles

Our results implied the existence of two independent behavioral dimensions to team communication. These are quantity of communication and quality, the latter being how effective this is in problem solving. However, we propose not using these names for behavioral dimensions, but rather the following: Social engagement (degree of communication involvement—how active was a person involved in the communication) and Personal agency/Communion (was this behaviour effective to solve job tasks or resolve social problems).

The team role is a particular behaviour pattern. From the team role perspective, *Social engagement* refers to how active a person is in communication with this team role. Some team roles have a high degree of communication involvement, e.g., *Initiator, Facilitator*. Other team roles, such as *Passive Collector* or *Outsider,* imply minimum communication with other team members, and therefore they can be understood as roles with low Social engagement (or high Social disengagement). Social sensitivity, discussed earlier, refers to the personal perception of social problems and cannot be used as a measure of communication involvement.

Personal agency-Communion defines behavioural orientation to social needs or to the project task completion. Personal agency is the ability of a person (actor) to put in effort to make things happen [53]. It represents what people believe and how they can regulate themselves to change the situation. Adapting this to the team role perspectives, Personal agency is a participant’s behaviour (team role) as they commit effort and perseverance to get a job done, i.e., a ’result-oriented’ team role. We propose that the opposite to Personal agency is Communion—a tendency to prioritise interpersonal relationship [54], or, in our case, team processes rather than individual actions. High communion means the role is more ‘team-oriented’ or ‘social-oriented’. In other words, team roles that are high in Communion help to solve social problems, whereas Personal agency helps get individual jobs done.

Furthermore, we propose that the team roles may be represented by a circumplex. The main principal of a circumplex is that variables (components) are arranged around a circle in two-dimensional space [55]. In general, a circumplex can be viewed from three different perspectives: as a pictorial representation, as a representation of circular order—components that are close to each other are more correlated and opposite components are negatively related—or as an exact circumplex structure when all components are equally spaced [56]. In our work, we understand a circumplex as a circular order of components. It is similar to the well-known ‘interpersonal circumplex’—a model for describing and organizing interpersonal behavior [57]. That model used a set of variables organized as a circle, and two dimensions—Dominance (personal agency) and Affiliation (Communion) [57,58]. 

**Figure 3:**
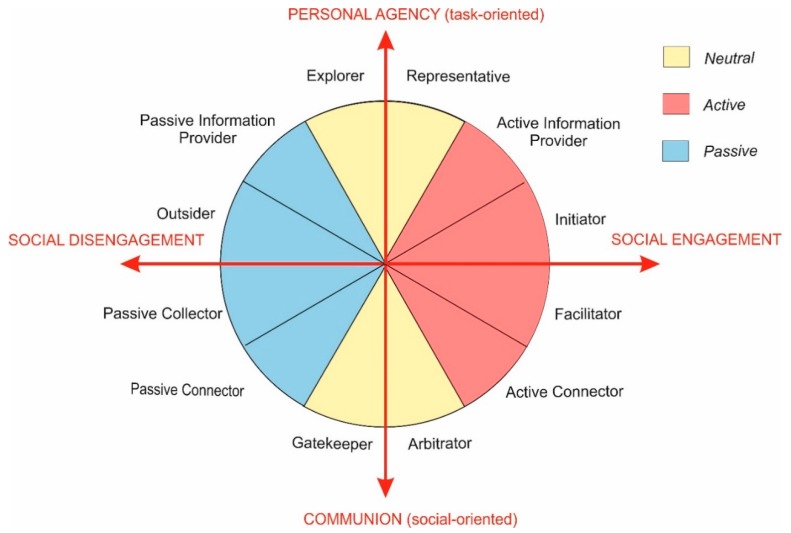
Circumplex of team roles.

In our study, however, we used Personal agency-Communion and Social engagement-Social disengagement as primary axes of the circumplex, as they better describe the team role behavioural dimensions. The elaborated roles, as per Nestsiarovich and Pons, may be used as a set of variables and put into a circular order around these axes. Each point within the circumplex represents a weighted mixture of Personal agency/ Communion and Social Engagement (see Figure 3).

We further propose that the roles can be categorized into groups of neutral, active, and passive. The blue colour on Figure 3 shows team roles with low social engagement (passive), red colour represents high social engagement (active), whereas yellow means neutral participation (middle level). 

Blue colour roles: *Passive Information Provider, Outsider, Passive Connector* and *Passive Collector.* We suggest calling them passive because they are low in Social engagement. People that chose these roles prefer being passive in communication (especially *Outsider* and *Passive Collector*). However, these roles involve different combinations of personal agency/communion*. Passive Information Provider* is more task than social-oriented because the person provides information only if somebody asks. *Outsider* merely ignores communication. P*assive Collector* is a little more involved in social processes by active listening and taking notes, whereas a *Passive Connector* has already a social task to complete (communicate with some person outside the group by emails).

Red colour roles: *Active Information Provider, Initiator, Facilitator* and *Active Connector.* The red colour in Figure 3 symbolizes team roles with high communication activity (Social engagement). An *Active Information Provider* gives information to the other team members: they explain something, answer questions, and help in problem-solving by providing missing details. *Initiators* are even more socially active, and suggest new ideas, show new directions, and are always ready for the new discussion to start. In this way, they may completely change the communication flow in the group. However, they are still more oriented on the task (project) problem-solving, rather than on social aspects, whereas a *Facilitator* not only helps to develop the project but also regulates communication processes in the team. This role is higher in Communion then *Initiator*.

Yellow colour roles: *Explorer, Representative, Arbitrator* and *Gatekeeper. Explorer* and *Representative* are high in Personal agency. These roles require quick task accomplishment, e.g., asking a question or answering the supervisor’s question when he/she addresses the whole group. Participants with a *Representative* role are more engaged in communication: if somebody asks a team about this part of the job, they represent a team and provide a response on behalf of the entire group. In contrast, *Explorer*s express only own opinions.

Finally, the roles of *Arbitrator* and *Gatekeeper* have goals to decrease the conflict level in the team and to invite passive members of the engineering team to contribute to the discussion. These are roles are both high in Communion. *Arbitrator* is a more socially engaged role because solving conflict may require a high level of communication activity. 

## 5. Conclusions

### 5.1. Outcomes 

This work developed a model that describes the process of team role assignment in project teams, analysed from the perspectives of personality (social sensitivity) and team needs. In addition, a circumplex of team roles was built to represent different aspects of these behaviour patterns and how team roles can be correlated with each other using Communion/ Personal agency and Social engagement axes.

### 5.2. Implication for Engineer Managers and Supervisors of Student Teams

The results of this work can be used by professionals in organisations and at university to build an effective team of engineers that can cope with complex projects by solving problems and having productive discussions during the project meeting time. We suggest the following:

1. First, sensitivity to team needs should be considered by people who are trying to build an effective project team of engineers: at least one person with high sensitivity in each team could be beneficial for project development. Team members with high levels of these parameters feel easier in conflict situations, and they generally try to take a team role that corresponds to team needs. This can be done by simple testing of potential team members, and by ongoing leadership of organisational culture;
2. The results of this study show that another important factor is participants’ satisfaction with team communication. People are happy with communication when chosen team roles are consistent with the individual objectives and personal preferences of participants. We suggest that team members could be given them an option to choose a team role according to their personality. For example, passive people may prefer to be *Passive Collectors* in project meetings, rather than *Facilitators*, and they should have a choice to behave according to their preferences. Managers or supervisors of the team can do this by testing potential or existing team members and finding the right place for them in a group or the right group. However, satisfaction also has to be balanced against (a) the project needs, and (b) personal growth. If team members only ever take roles in which they are comfortable, then their personal development would seem precarious. The circumplex may help here, by identifying adjacent roles that it may be easier for them to transition to;
3. Leadership of teams, which relates more to shaping people’s behaviour than management of project objectives, is identified with the Yellow colour roles of *Explorer, Representative, Arbitrator* and *Gatekeeper*. A key aspect of engineering team leadership appears to be the ability to solicit contributions from quieter members and facilitate, but not dominate, the discussion. At the next level in the organisation, leadership involves shaping the organisational culture to encourage behaviours that enhance team performance, and the personal development of subordinates;
4. The circumplex of team roles could be used to analyse a balance of passive and active behavioural patterns in a team. It is a visual representation of team communication activity and role distribution in a group: what kind of communication behaviour is the most typical for the team, which role is missing, how active team members are in discussion project problems. According to our study results, the high activity of team members does not guarantee project success. Even very passive communication teams still have a chance to complete a project successfully. However, we assume that the chance for project success increases if a team has at least one active team member willing to discuss a problem and to coordinate others. If not, team members may find that their meetings are less productive than they could be, and hence may need to spend more time in discussion.

### 5.3. Limitations 

This work has several limitations:

- Supervisors of student teams followed their official position duties and it was hard to identify their real preferences in communication style;
- Agreeableness can be taken only as an approximate measure of social sensitivity;
- Observations were conducted on students at their official meetings with supervisor or clients, whereas students may have other types of meetings between each other that were not observed;
- The division between main and secondary team roles should be considered a rough approximation, as sometimes it was hard to see the difference;
- Gender, age and other demographic factors were not considered in this study.

### 5.4. Future Research Questions

- A bigger sample and more statistical data could give additional information about correlations between personality and team roles;
- Factors that influence team communication, such as the presence/absence of supervisor, location of the meeting, relationship between participants and style of supervision, need further study;
- Other personality traits (Extroversion, Conscientiousness, Openness to experience) could be correlated with the team role choice, too;
- Finally, it could be interesting to compare the team role assignment in an engineering team at university and in a commercial firm.

### 5.5. Original Contributions

The work makes the following original contributions. First, it provides a procedure to analyze the choice of group roles in accordance with such parameters as social sensitivity, personal and team needs. Second, a circumplex was built to represent the variety of team roles from the perspective of Social engagement and Communion/personal agency.

## Figures and Tables

**Table 1:** Team roles per Nestsiarovich and Pons. Reproduced from [33] by permission.

*N*	Team Roles	Typical Communication Pattern
1	*Initiator* (initiate process)	Active participation, propose new ideas and tasks, as well as new directions of work.
2	*Passive collector* (collect information)	Passive data collecting, non-verbal signs of agreement or just short yes/no answer, low verbal participation in team discussion, attentive listening, and keeping ideas inside (non-vocalisation).
3	*Explorer* (ask questions)	High verbal participation, active data collecting: ask general questions, ask for different facts, ideas or opinions, and explore facts. Ask to clarify or specify ideas, define the term, and give an example.
4	*Information provider* (provide information)	Provide detailed and excessive information: take an active part in the conversation, but mostly talk rather than listen.
5	*Facilitator* (summarise, control discussion)	Define the task or group problem, suggest a method or process for accomplishing the task, provide a structure for the meeting, control the discussion processes. Bring together related ideas, restate suggestions after the group has discussed them, offer a decision or conclusion for the group to accept or reject. Get the group back to the track.
6	*Arbitrator* (solve disagreement)	Encourage the group to find agreement whenever a miscommunication arises, or group cannot come to a common position.
7	*Representative* (express, answer)	Verbalise group’s feelings, hidden problems, questions or ideas that others were afraid to express, provide an answer to questions that were referred to the whole group.
8	*Gatekeeper* (fill gaps, sensitive to others)	Help to keep communication channels open: fill gaps in conversation, ask a person for his/her opinion, be sensitive to the non-verbal signals indicating that people want to participate.
9	*Connector* (connect people)	Connect the team with people outside the group.
10	*Outsider* (stay outside)	Do not participate in project discussion.

**Table 2:** Statistical data from Question 6.

Team role	Quantity per Teams 1–5
Initiator	16
Passive collector	9
Explorer	14
Information Provider	9
Facilitator	14
Arbitrator	9
Representative	9
Gatekeeper	13
Connector	7
Outsider	1

**Table 3:** Team roles of the student teams 1–5.

Participant	Main Role 1	Main Role 2	Secondary Role 1	Secondary Role 2
1A	Explorer	Initiator	Representative	Passive Connector
1B	Passive Collector	Passive IP ^1^	-	-
1C	Facilitator	Explorer	Passive IP	Representative
1D	Explorer	Explorer	Initiator	Gatekeeper
**1E**	**Facilitator**	**Initiator**	**Active IP**	**Explorer**
2A	Explorer	Active IP	Gatekeeper	Representative
2B	Representative	Representative	Active IP	Gatekeeper
2C	Representative	Gatekeeper	Passive Collector	Explorer
2D	Passive Collector	Representative	Explorer	Gatekeeper
**2E**	**Facilitator**	**Passive IP**	**Passive Collector**	**Explorer**
3A	Passive Collector	Passive Collector	Passive IP	Outsider
**3B**	**Facilitator**	**Explorer**	**Passive IP**	**Initiator**
3C	Passive Collector	Representative	Arbitrator	Explorer
3D	Active IP	Passive Collector	Connector	Explorer
3E	Initiator	Representative	Explorer	Arbitrator
4A	Explorer	Active IP	Initiator	Gatekeeper
4B	Facilitator	Initiator	Active Connector	Passive IP
4C	Explorer	Initiator	Passive Collector	-
4D	Explorer	Representative	Active Connector	Initiator
**4E**	**Facilitator**	**Passive Collector**	**Passive IP**	**-**
**5A**	**Facilitator**	**Explorer**	**Passive IP**	**Active IP**
5B	Passive Collector	Passive Collector	Explorer	Passive IP
5C	Facilitator	Gatekeeper	Passive Collector	Active IP
5D	Facilitator	Active IP	Representative	Initiator
5E	Initiator	Gatekeeper	Facilitator	Active IP

^1^IP—Information Provider. Bold type denotes supervisor

**Table 4:** Team role assignment in student teams 1–5.

Team	Team Needs	Partici-Pant	Agree-Ableness	Personal Attitude	Main Team Roles	Secondary Team Roles
1	Gatekeeper *‘Disengagement of some team members’* Connecter Communication problems with a client	1A	40	good	Explorer Initiator	Representative Passive Connector
		1B	17	good	Passive Collector Passive IP	-
		1C	51	unhappy	Facilitator Explorer	Passive IP Representative
		1D	45	very happy	Explorer	Initiator Gatekeeper
		**1E**	**71**	**happy**	**Facilitator** Initiator	Active IP Explorer
2	Arbitrator, Facilitator *‘Lack of communication when problems arise’* Information Provider *‘Sometimes lack specific details to truly enable progress’* Gatekeeper Some team members were regularly prevented from talking by other too active participants; *‘lack of engagement from some team members’*	2A	21	satisfied	Explorer Active IP	Gatekeeper Representative
		2B	14	good	Representative Gatekeeper	Active IP -
		2C	76	good	Representative	Passive CollectorExplorer
		2D	40	happy	Passive Collector Representative	ExplorerGatekeeper
		**2E**	**83**	**good**	Facilitator Passive IP	Passive Collector Explorer
3	Facilitator, Arbitrator *‘Team was not very good at planning early’* *‘A lot of time was wasted on things we did not think was necessary’*	3A	2	satisfied	Passive Collector	Passive IPOutsider
		**3B**	**30**	**unhappy**	Facilitator Explorer	Passive IP Initiator
		3C	67	good	Passive Collector Representative	Arbitrator Explorer
		3D	35	good	Active IP Passive Collector	Connector Explorer
		3E	40	good	InitiatorRepresentative	Explorer Arbitrator
4	Passive Collector, Gatekeeper *‘Too much people talking at once’. ‘One person talks too much. Other person is reluctant to put their ideas formed’.* Some students regularly monopolized talking time	4A	45	happy	Explorer Active IP	Initiator Gatekeeper
		4B	83	happy	Facilitator Initiator	Active Connector Passive IP
		4C	80	very happy	Explorer Initiator	Passive Collector -
		4D	56	very happy	Explorer Representative	Active Connector Initiator
		**4E**	**67**	**happy**	Facilitator Passive Collector	Passive IP
5	Initiator, Explorer Lack of active interactions between participants	**5A**	**67**	**happy**	Facilitator Explorer	Passive IP Active IP
		5B	30	unhappy	Passive Collector	Explorer Passive IP
		5C	71	good	Facilitator Gatekeeper	Passive Collector Active IP
		5D	21	happy	Facilitator Active IP	Representative Initiator
		5E	51	very happy	Initiator Gatekeeper	Facilitator Active IP

Green colour highlights a team role that corresponds to team needs. Bold type represents supervisors of the team.

**Table 5:** Social sensitivity and satisfaction in the project team.

Social sensitivity	Satisfaction in the team	
	Low satisfaction *(unhappy, satisfied)*	High satisfaction *(happy, very happy)*
High social sensitivity (*Agreeableness* over 50)	**Reluctant cohesiveness** Participants may accept a role that is needed in his team; however, they are not happy with the communication processes because their roles are not consistent with their individual objectives and expectations.	**Team coherence** Participants may accept the role that team needs and this is consistent with their individual objectives and expectations, so they feel happy.
Low social sensitivity (*Agreeableness* below 35)	**Behavioural divergence** Participants follow their own ideas and preferences in communication, however there are some problems in the team (or personal problems) that cannot be solved by this.	**Parallel compensation** Participants follow their own ideas and preference in communication, and this apparently makes them feel satisfied with the team communication. However, the operational needs of the team may not be met.

## Appendix A. Initial Questionnaire for Students

1. Please identify your gender__________
2. What engineering discipline do you study? _________________
3. What is the level of your highest qualification? _______________
4. To what extent do you know other team members:
  - I do not know anybody
  - I can recognise several members of this group
  - I know one-two members of this group very well and recognise others
  - I know several members of this group very well and recognise others
  - I know all team members very well

## Appendix B. Interview Questions

1. How comfortable did you feel in this team communication? *(please use scale from 0–10) *
  ________________________________________________________________________________________________________________________________________________________
  What did you like? What was wrong? _______________________________________________________________________________________________________________________________________________________________________________________________________________________________________________________________
2. **[students only]** Please estimate your contribution to the project? *(please use scale from 0–10) __________________*
3. To what extent did you feel that miscommunication occurs in your meetings? *[never, sometimes, most times, always].* What do you think were the typical causes for this? _____________________________________________________________________________________________________________________________________________________
4. How productive do you think was your team in problem-solving? *(please use scale from 0–10)__*
  According to you, what were the barriers for team productivity and successful problem-solving? And what were the strong aspects of communication in your team? _____________________________________________________________________________________________________________________________________________________________________________________________________________________________________________________
5. What is your intuitive perception of your own communication style in this team?
  ________________________________________________________________________________________________________________________________________________________________________
6. Please tick all team roles (communication patterns) that you think describe your typical communication behaviour?
  - *Initiator(Initiate process)*—Active participation, propose new ideas and tasks, newdirectionsof work.
  - *Passive collector(Collect information)* —Passive data collecting, non-verbal signs of agreement or just short yes/no answer, low verbal participation in team discussion, attentive listening, and keeping ideas inside.
  - *Explorer(Ask questions)*—High verbal participation, active data collecting: ask general questions, ask for different facts, ideas or opinions, and explore facts. Ask to clarify or specify ideas, definetheterm, and give an example.
  - *Information provider*—Provide detailed and excessive information: takeanactivepart intheconversation, but mostly talk than listen
  - *Facilitator(Summarize, control discussion)*—Define the task or group problem; suggest a method or process for accomplishing the task; provide a structure for the meeting, control the discussion processes.Bring together related ideas, restate suggestions after the group has discussed them, offer a decision or conclusion for the group to accept or reject. Get the group back to the track
  - *Arbitrator (Solve**disagreement*) —Encouragethegroupto find agreement whenever miscommunicationarises, orgroup cannot come to the common division.
  - *Representative(Express, answer)*—Verbalize group’s feelings, hidden problems, questions or ideas that others were afraid to express, provideananswerto the question that referred to all group.
  - *Gatekeeper* (*Fill gaps, sensitive to others*)—Help to keep communication channels open, fill gaps in conversation, ask a person for his/her opinion, be sensitive to the non-verbal signals indicating that people want to participate.
  - *Connector* (*Connect*)—Connect the team with people outside the group
  - *Outsider*—Stay in the room but do not participate in project discussion (think about something else)
7. Do you feel that you changed your communication behaviour at different meetings? Which communication situations caused that? __________________________________________________________________________________________________________________________________________________________
8. To what extent do you feel that other people’s discussions prevented you from making a contribution at meetings? ________________________________________________
9. **[students only]** If you happened to be elected Team Leader at some meeting, did you feel comfortable in this role? If not, why not?
  ________________________________________________________________________________________________________________________________________________________
10. **[students only]** Do you feel more comfortable at meetings to address your ideas to other students rather than to supervisor and client? Why is that? ________________________________________________________________________________________________________________________________________________________________________
11. If you need to say something, which situation is more natural for you: to talk with a particular person or to transmit ideas to the whole team? ___________________________________________________________________________________________________________________________________________________________________
12. What is your preferable style of communication at meeting: slow but accurate discussion, middle intensity of communication, or communication at high speed with quick exchanging of ideas? According to you, which meeting style is the most helpful in problem-solving? ________________________________________________________________________________________________________________________________________________________________________
  Which style of communication ‘students- supervisor’ at project discussions do you prefer? (extensive freedom, less freedom, total control). Do you think it predefines the results of project performance? Why? ________________________________________________________________________________________________________________________________________________________
13. Did you feel that location of the meeting and your position inside the room predefines your communication style? What position was the most comfortable for you? ______________________________________________________________________________________________________________________________________________________
14. Sometimes people in meetings make use of physical objects like drawings, papers, computer screens, physical models, whiteboard drawing, etc. To what extent did you find it helpful when people presented these types of objects? *[never, sometimes, most times, always]*. Why do you think so? Are there situations where these objects were distracting or caused miscommunication? __________________________________________________________________________________________________________________________________________________________________________________
